# Probiotic potentials of *Lactobacillus plantarum* isolated from fermented durian (Tempoyak), a Malaysian traditional condiment

**DOI:** 10.1002/fsn3.672

**Published:** 2018-06-02

**Authors:** Asmariah Ahmad, Wei Boon Yap, Noorhisham Tan Kofli, Ahmad Rohi Ghazali

**Affiliations:** ^1^ Programme of Biomedical Science and Centre for Health and Applied Sciences Faculty of Health Sciences Universiti Kebangsaan Malaysia (UKM) Kuala Lumpur Malaysia; ^2^ Department of Chemical & Process Engineering Faculty of Engineering & Built Environment Universiti Kebangsaan Malaysia Bangi Selangor Malaysia

**Keywords:** antiproliferation, *Lactobacillus plantarum*, Malaysian traditional condiment, probiotic, tempoyak

## Abstract

Lactic acid bacterium isolated from fermented durian (tempoyak) was investigated for its potentials as a probiotic strain. Bacterial tolerance toward gastrointestinal environment, adhesion, and cytotoxic activity in human colon adenocarcinoma cell line HT‐29 was evaluated. 16S rRNA sequencing identified the lactic acid bacterium as *Lactobacillus plantarum*. The bacterium demonstrated good tolerance toward gastrointestinal pH 2.0 and 0.3% bile salts. It showed strong adhesive capacity in human intestinal cell line, HT‐29, with an adhesion index of 159 ± 10. Cytotoxicity of *L. plantarum* was investigated using both live bacterial cells (BC) and cell‐free supernatant (CFS). Findings showed that both BC and CFS of *L. plantarum* reduced proliferation of HT‐29 colon adenocarcinoma cells using MTT assay. The results imply potential probiotic properties of *L. plantarum* isolated from tempoyak.

## 1 INTRODUCTION

The idea of using probiotics to promote optimal health and help reduce risk of diseases has been an interesting subject for several decades. First postulated by Ellie Metchnikoff in early 1900s, a theory that bacillus isolated from Bulgarian *kiselo mleko* could colonize intestinal tract and contribute to the health of human beings was made following his observation on the longevity of Bulgarian peasants that was largely associated with the consumption of fermented milk products. The Nobel Laureate proposed that by manipulating the intestinal microbiome with host‐friendly bacteria found in yogurt could lead to health‐enhancing and delayed‐senility effects (Mackowiak, 2013). Throughout the years, many definitions of probiotics have been proposed by researchers. Some defined probiotics as compounds that either stimulate bacterial growth or improve host immunity (Fujii & Cook, 1973) while other defined them as organisms and substances which contribute to intestinal microbial balance (Parker, 1974). FAO/WHO (2002) then suggested the definition of probiotics as live microorganisms that when administered adequately can boost its host's health.

Probiotics are beneficial bacteria that maintain intestinal microflora balance, inhibit the growth of harmful bacteria, promote good digestion, increase resistance to infection, and boost immune functions (Helland, Wicklund, & Narvhus, 2004). Other physiological benefits of probiotics include removal of carcinogens from the intestinal tract, immunomodulation of host immune system by reducing allergic reactions, and lactose intolerance, as well as to enhance nutrient bioavailability in hosts (Parvez, Malik, Ah, & Kim, 2006). Most probiotic microorganisms belong to the lactic acid bacteria (LAB) group, such as *Lactobacillus* spp. and *Enterococcus* spp. or genus *Bifidobacterium* (Klein, Pack, Bonaparte, & Reuter, 1998). LAB are commonly found in fermented products and have been widely used as starter cultures in food industry (Carr, Chill, & Maida, 2002). Some LAB such as *Lactobacillus* and *Bifidobacterium* strains are widely available as commercial probiotics (Jamaly, Benjouad, & Bouksaim, 2011). Given various members of LAB, thorough screening and characterization of LAB are, therefore, important before considering them as potential probiotic strains. The selection criteria for probiotic LAB include ability to survive in the gastrointestinal tract environment such as gastric and bile acid conditions, resistance to antibiotics, and adhesion to gastrointestinal epithelial lining (Pisano et al., 2014). Additionally, a good probiotic candidate must possess desirable physiological criteria such as antagonism toward microbial pathogens, cholesterol metabolism, antimutagenic, and anticarcinogenic properties in hosts (Vasiljevic & Shah, 2008).

Tempoyak is a traditional fermented condiment made from the pulp of durian in Malaysia and Indonesia. Durian flesh is mixed with salt (2.5%, w/v), placed in a sealed container, and allowed to ferment for 1 week. Tempoyak is often used as condiment with certain fish and vegetable dishes (Endo, Irisawa, Dicks, & Tamasupawat, 2014). Tempoyak typically has long shelf life because it is preserved by lactic acid produced by LAB and salt added during processing to inhibit growth of food‐spoiling bacteria (Amiza, Zakiah, Ng, & Lai, 2006). Earlier analysis showed that LAB are the most predominant microorganisms that present in tempoyak. In addition, several organic acids such as acetic, lactic, and propionic acids were also detected (Chuah et al., 2016). *Lactobacillus* spp., *Leuconostoc* spp., and *Fructobacillus durionis* are among the known LAB that had been isolated from tempoyak (Endo et al., 2014) with *Lactobacillus plantarum* and *F. durionis* as the dominant LAB members (Chuah et al., 2016; Leisner et al., 2001). The chemical compositions of durian flesh with a total sugar content of 15%–20% and saccharose of 17% are nutritious enough to support the growth of LAB (Ketsa & Daengkanit, 1998; Leisner et al., 2001). Despite its popularity as one of the Malaysian traditional fermented condiments, very limited research has been conducted on the health benefits of tempoyak, especially its LAB content. Thus, the objective of our study was to identify and characterize LAB strain isolated from tempoyak in accordance with the established criteria and hence a potential probiotic strain in local food industry.

## 2 MATERIALS AND METHODS

### 2.1 Isolation and preparation of LAB

Lactic acid bacteria was provided by the Department of Chemical and Process Engineering, Faculty of Engineering and Built Environment, the National University of Malaysia. Tempoyak purchased from market was briefly plated on MRS agar (de Man, Rogosa and Sharpe Oxoid, Thermo Fisher). The plates were incubated at 37°C for 48 hr. Single colonies were picked and streaked onto MRS agar and then subcultured up to three times for bacteria purification. Isolated colonies were subjected to Gram staining, catalase test, and genetic identification using 16S rRNA. The isolated colonies were further cultured in MRS broth (Laboratorios Conda, Spain) and kept in 20% (v/v) glycerol until further use. Bacterial isolates were grown on MRS agar and in MRS broth prior to assays. Bacteria were grown in MRS broth at 37°C for 16–18 hr. The whole culture (WC) bacterial samples were then prepared in two forms, bacterial cells (BC) and cell‐free supernatant (CFS). Both BC and CFS were collected from freshly grown bacterial culture at 10,000 *g* for 10 min; the pellet was then washed, centrifuged, and resuspended in 0.1 mol/L phosphate buffer saline (PBS) as BC and CFS were obtained after supernatant from freshly grown culture was filter‐sterilized (0.22 μmol/L pore size; Sartorius).

### 2.2 Lactic acid bacteria identification by 16S rRNA sequences

Molecular identification of the bacterial isolates was made by 16s rRNA gene sequencing (First Base Laboratories SDN. BHD). Two universal primers of 518F (5′ CCAGCAGCCGCGGTAATACG 3′) and 800R (5′ TACCAGGGTATCTAATCC 3′) were used in order to amplify 16S rRNA gene in a polymerase chain reaction (PCR). The amplified 16S rRNA gene sequence was subsequently compared with the GenBank database using Basic Local Alignment Search Tool (BLAST).

### 2.3 Antibacterial activity

The inhibitory potential of bacterial isolates against pathogenic bacteria was evaluated with well diffusion assay using WC, BC, and CFS. The pathogenic bacteria used as indicators strain were gram‐negative *Escherichia coli* ATCC25922 and gram‐positive *Staphylococcus aureus* ATCC25923. Briefly, 50 μl of bacterial suspension of indicator strains (10^8^ CFU/ml, equivalent to 0.5 McFarland standard) was spread on Mueller Hinton agar plates (Oxoid Ltd, Hampshire, UK) and allowed to dry. Three wells (6 mm, in diameter, each) were made with sterile borer. Then, 20 μl of WC, BC, and CFS of *L. plantarum* was added separately into the wells. The plates were incubated at 37°C for 24 hr. The diameter of zone of inhibition was measured and expressed as mean ± standard deviation (*SD*).

### 2.4 Acid tolerance activity

In acid tolerance assay, bacteria (100 μl) collected from overnight‐grown cultures were transferred into 900 μl PBS adjusted to pH 1.5, 2, 3, and 4 and then incubated at 37°C. The numbers of viable bacteria were determined at 0 and 3 hr of incubation, on MRS agar plate. The assay was performed in triplicate. Data obtained from the study were expressed in terms of log_10 _CFU/ml and as mean ± *SD*.

### 2.5 Bile salts tolerance activity

In the bile tolerance assay, 100 μl of overnight culture was transferred into a 900 μl of MRS broth containing 0.3% (w/v) bile salt (Oxoid, Thermo Fisher) and then incubated at 37°C. The number of viable bacteria was determined at 0 and 3 hr by incubating the cells on MRS agar. The assay was carried out in triplicates. Data were expressed in terms of log_10 _CFU/ml and as mean ± *SD*.

### 2.6 Antioxidant activity assays

#### 2.6.1 DPPH‐free radical scavenging assay

Turbidity of WC and BC interferes with absorbance reading; therefore, CFS alone was tested in the assay. The DPPH‐free radical scavenging assay of CFS of *L. plantarum* was measured according to Afify, Romeilah, Sultan, and Hussein (2012) with slight modifications. Briefly, 1 mmol/L DPPH solution in ethanol was prepared, and 50 μl of the solution was added to 950 μl of CFS or ascorbic acid (positive control) and incubated for 30 min at room temperature. DPPH solution added with MRS broth served as negative control. The results were read at 517 nm. The percentage of radical scavenging activity was calculated according to the following equation; A0 = negative control, A1 = Sample

$$\text{Scavenging activity}\left(\%\right)=\left[\frac{A0-A1}{A0}\right]\times 100$$

#### 2.6.2 Ferric‐reducing ability of plasma (FRAP) assay

Ferric‐reducing ability of plasma assay was conducted according to Benzie and Strain (1996) with minor modifications, and antioxidant capacity of CFS of *L. plantarum* was measured based on its ability to reduce ferric tripyridyltriazine [Fe (III)‐TPTZ] complex to ferrous‐tripyridyltriazine [Fe (II)‐TPTZ]. The FRAP reagents were prepared at 1:1:10 ratio in which contained 10 mmol/L of TPTZ solution in 40 mmol/L of HCl, 20 mmol/L FeCl_3_, and 0.3 mmol/L of acetate buffer with pH 3.6. About 50 μl of sample or standard was mixed with 175 μl of FRAP reagent and incubated at 37°C for 5 min. The result was measured at 595 nm. FeSO_4_ solution at various concentrations was used as standard, and ascorbic acid was used as positive control.

### 2.7 Cell culture

HT‐29 (ATCC 38‐HTB) colon adenocarcinoma cell line was purchased from American Type Culture Collection (Rockville, MD, USA). The cell line was maintained in McCoy's 5A medium (McCoy's 5A; Gibco, USA) at 37°C under 5% CO_2_ environment until 80%–90% confluency. For MTT assays, cells were seeded in 96‐well tissue culture plates (NEST, China), whereas for adhesion assays, cells were prepared on glass coverslips placed in six‐well plates (NEST).

### 2.8 Antiproliferation activity

Cell proliferation was assayed using MTT kit (Thirabunyanon & Hongwittayakorn, 2013) 3‐(4, 5‐dimethylthiazole‐2‐yl)‐2, 5‐diphenyl tetrazolium (MTT; Sigma). Cells were seeded and cultured on 96‐well tissue culture plates at 5 × 10^4^ cells/ml, 37°C for 24 hr. In this assay, BC and CFS were tested separately in order to justify the biological active component of antiproliferative activity. After incubation, 100 μl of BC or CFS was added to each well and further incubated for 24 hr. Cells added with MRS and PBS were prepared as negative controls. Cells were then washed thrice with PBS. About 20 μl of MTT solution (5 mg/ml in PBS, pH 7.4) was added and incubated further for 4 hr. Formazan crystals were then solubilized with dimethyl sulfoxide (Thermoscientific) and further incubated for 15 min. The intensity was determined with an ELISA reader (Bio‐Rad, USA) at 570 nm. The percentage of viable cells was calculated by the following equation:

$$\text{Cell viability}\;\left(\%\right)=\left[\frac{\text{Sample}\;\text{O.D}}{\text{Control}\;\text{O.D}}\right]\times 100$$

### 2.9 In vitro adhesion assay

Adherence of bacteria to HT‐29 was examined as described previously by Gopal, Prasad, Smart, and Gill (2001) with a few modifications. Monolayer of HT‐29 cells was prepared on glass coverslip placed in a six‐well tissue culture plate at a 5 × 10^4^ cells/ml. The assay required HT‐29 cells achieve 90%–100% confluency. Prior to assay, the monolayer was washed thrice with PBS. BC at 10^8^ CFU/ml were added to the monolayer on glass coverslips in six‐well tissue culture plate in the presence of McCoy's 5A without antibiotic–antimycotic solution. The plate was then incubated for 1 hr in 5% CO_2_ at 37°C. The monolayer was washed thrice with PBS and fixed in ice‐cold 3:1 methanol‐acetic acid fixative for 15 min. After fixing, the monolayer was washed thrice with PBS and allowed to air‐dry and be Gram‐stained. The coverslip was then examined microscopically under oil immersion lens. The number of adhered bacteria on 20 randomized microscopic fields was enumerated and presented as mean ± *SD*.

### 2.10 Scanning electron microscopy (SEM)

For SEM analysis, HT‐29 colon adenocarcinoma cell line was prepared as described in the previous section and fixed with 2.5% (v/v) glutaraldehyde in 0.1 mol/L phosphate buffer at room temperature. The cells were then washed thrice with PBS, followed by dehydration in increasing concentrations of ethanol 30, 50, 70, 80, 90 (10 min per step), and 100% (v/v) (thrice and 10 min each). Cells were dried in a critical point drier (Leica EM 300, Germany) and coated with gold. The specimens were then examined with LEO 1450VP scanning electron microscope at Microscopy Electron Unit, Faculty of Science and Technology, Universiti Kebangsaan Malaysia.

### 2.11 Statistical analysis

All data were expressed as mean ± *SD*. For statistical analysis, SPSS version 20 (IBM, USA) was used. One‐way ANOVA was used to analyze the data, followed by Tukey's test for post hoc comparisons. Data with *p* < .05 were considered statistically significant.

## 3 RESULTS AND DISCUSSION

**Figure 1:**
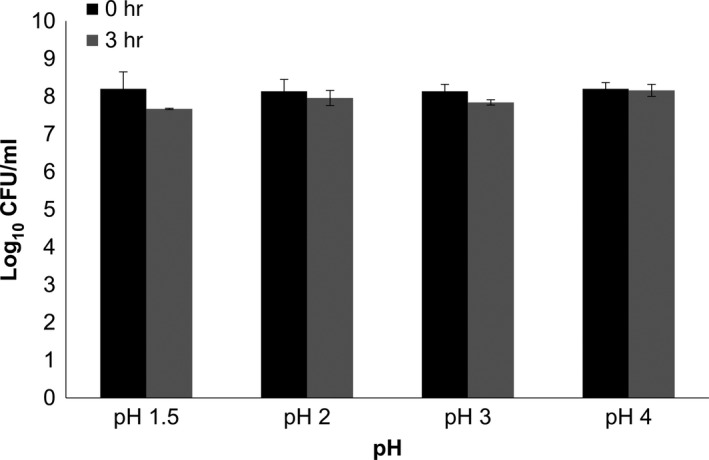
Acid tolerance activity of bacterial isolate on various gastrointestinal pH for 0 and 3 hr. Values were presented as log_10_ CFU/ml in mean ± standard deviation of three replications

**Figure 2:**
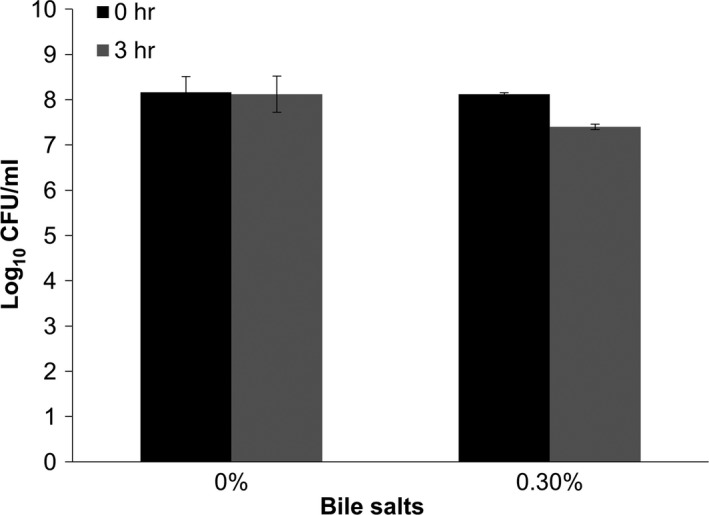
Tolerance of bacterial isolate toward 0.3% of bile salt solution for 0 and 3 hr. Values were presented as log_10_ CFU/ml in mean ± standard deviation of three replications

In the present study, probiotic and cytotoxic potentials of a lactic acid bacterium isolated from tempoyak (fermented durian) were investigated using in vitro model. Its tolerance toward gastrointestinal environment, antimicrobial activity, antioxidant activities, adhesion capacity, and antiproliferative effects on colon adenocarcinoma cell line was determined. The isolate was molecularly identified as *L. plantarum* by 16S rRNA sequencing. The isolate showed high tolerance toward acidic (Figure 1) and bile gastrointestinal environment (Figure 2). Similarly, Lee, Bong, Lee, Kim, and Park (2016) reported that *L. plantarum* isolated from kimchi, a Korean traditional fermented cabbage, showed reasonable survivability after 3 hr of exposure to pH 3.0 and 0.3% bile salts. Table 1, on the other hand, shows that both CSF and WC of *L. plantarum* inhibited the growth of indicator bacteria in the antimicrobial assay. However, there was no inhibition by BC thus implying that bacterial metabolites and secretions in the medium were in fact responsible for the antimicrobial activities. Among soluble antimicrobials produced by the majority of LAB include bacteriocins, organic acids, and diacetyl (Con & Gokalp, 2000).

**Table 1:** Antimicrobial activity of *Lactobacillus plantarum* against pathogenic indicator strains

*L. plantarum*	Inhibition zone (mm)	
	*Escherichia coli* (ATCC 25922)	*Staphylococcus aureus* (ATCC 25923)
WC	3.9 ± 0.1	10.3 ± 0.64
CFS	3.9 ± 0.05	10.1 ± 0.36
BC	–	–

WC, whole culture; BC, bacterial cells; CFS, cell‐free supernatant.

Data are presented as diameter of zones of inhibition (mm) as mean with standard deviation of three replications.

Cell‐free supernatant of *L. plantarum* exhibited lower scavenging activity (14.4%) toward DPPH‐free radical as compared to ascorbic acid (68.4%) (Table 2). Meanwhile, antioxidant capacity of CSF of *L. plantarum* was 41.08 μmol/ml as compared to ascorbic acid (76.12 μmol/ml) (Table 3) in the FRAP assay. According to Rjiniemon, Hussain, and Rajamani (2015), the antioxidative properties of LAB isolated from traditional fermented foods could be potentially useful in treating chronic diseases such as cancer and diabetes. The study also found that *L. plantarum* isolated from fermented ragi malt was able to scavenge DPPH‐free radicals. Findings from this study also support observation by Nyanzi, Shuping, Jooste, and Eloff (2015) in which *Lactobacillus* strains showed various DPPH scavenging effect, ranging from low to high scavenging rate. For instance, *Lactobacillus acidophilus* showed 11.4%–86.7% scavenging rate meanwhile *Lactobacillus rhamnosus* only showed 4.9%–66.9% scavenging rate. This implies that antioxidant activity of *Lactobacillus* spp. varies among species and strains. The antioxidative activity of *Lactobacillus* spp. is mostly contributed by exopolysaccharides (EPS) (Ghalem, 2017; Zhang et al., 2012).

**Table 2:** Scavenging effects of cell‐free supernatant (CFS) of *Lactobacillus plantarum* and positive control (ascorbic acid) on DPPH‐free radicals

Source of extracts	% Inhibition
*L. plantarum* (CFS)	14.5 ± 0.1
Ascorbic acid (Vit C)	68.4 ± 0.02

Values are represented as mean ± standard deviation (*n* = 3).

**Table 3:** Antioxidant capacity of cell‐free supernatant (CFS) of *Lactobacillus plantarum* and positive control (Vit C)

Source of extracts	FRAP value (μmol/ml)
*L. plantarum* (CFS)	41.08 ± 2.7
Ascorbic acid (Vit C)	76.12 ± 3.7

FRAP, ferric‐reducing ability of plasma.

Values are represented as mean ± standard deviation (*n* = 3).

According to Kim, Woo, Kim, Kim, and Lee (2003), different fractions of LAB, such as whole cells, heat‐killed cells, the cell wall, peptidoglycan, and cytoplasmic fraction, have inhibitory effect against human cancer cell lines. Wang et al. (2014) reported that *Lactobacillus* strains isolated from fermented food showed significant antiproliferative and apoptosis effects in HT‐29 colon adenocarcinoma cell line but remained harmless to noncancerous Vero kidney cell line, implying that LAB possess selective toxicity on cancer cells. In another study by Tuo et al. (2010), heat‐killed bacteria cells, cell wall, and genomic DNA of wild *Lactobacillus* strains isolated from various fermented foods in China were able to inhibit proliferation of HT‐29 cell line. Meanwhile, Thirabunyanon, Boonprasom, and Niamsup (2009) also found that both cell medium and live WC of probiotic bacteria isolated from fermented dairy product in Thailand, namely *Enterococcus faecium* RM11 and *Lactobacillus fermentum* RM28 inhibited Caco‐2 colon adenocarcinoma cell growth. In the present study, we investigated the antiproliferative properties of the BC and CFS of *L. plantarum* on human colon adenocarcinoma cell line HT‐29.

**Figure 3:**
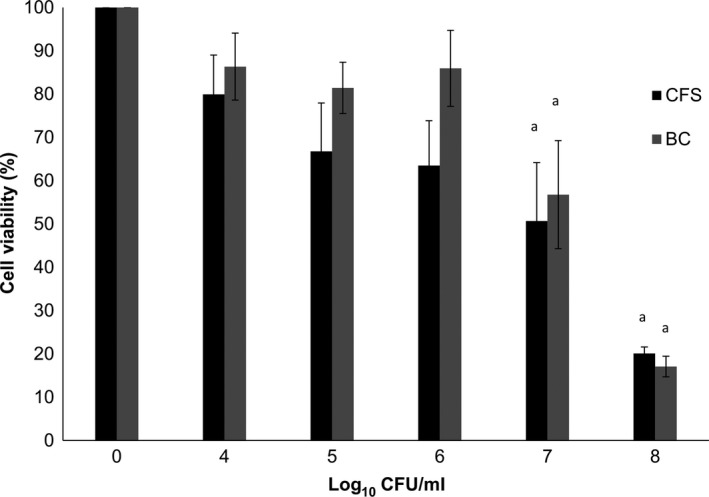
Proliferation of HT‐29 colon adenocarcinoma after 24‐hr incubation with cell‐free supernatant (CFS) and bacterial cells (BC) at different cell concentrations. The results were determined using MTT assay kit. Values are presented as mean ± *SE* of three independent experiments. ^a^Significant (*p* < .05) in relative to control

Bacterial cell and CFS of *L. plantarum* isolated from tempoyak showed a dose‐dependent cytotoxicity in HT‐29 cells as detected by the MTT assay (Figure 3). CFS exhibited stronger inhibition on the cancer cells than BC. This is in parallel to the findings reported by Er, Koparal, and Kivanc (2015) that *Pediococcus pentosaceus*,* L. plantarum* and *Weissella confusa* isolated from fermented meat were able to inhibit the growth of colon cancer cells in a dose‐dependent manner and, more interestingly, cell‐free filtrate of *L. plantarum* also showed stronger effects compared with the other two strains. Our findings are also in accordance with a previous study by Haghshenas et al. (2015) which demonstrated that secreted metabolites of *L. plantarum* 17C isolated from ewe colostrum exhibited antiproliferative effect on HT‐29 cell line. Antiproliferative activity of probiotic strains on colon cancer cells could be due to the presence of EPS (Sadeghi‐Aliabadi, Mohammadi, Fazeli, & Mirlohi, 2014). According to Kim et al. (2006), cell‐bound EPS (cb‐EPS) and released EPS (r‐EPS) of *L. rhamnosus* inhibited the growth of HT‐29 and PANC‐1 pancreatic cancer cells. When comparing the two types of EPS, r‐EPS exhibited stronger cytotoxic effects than cb‐EPS. The ability of probiotic bacteria to exert cytotoxicity effects on colon cancer cells thus suggests its potential to be developed as chemopreventive for colon cancer.

**Figure 4:**
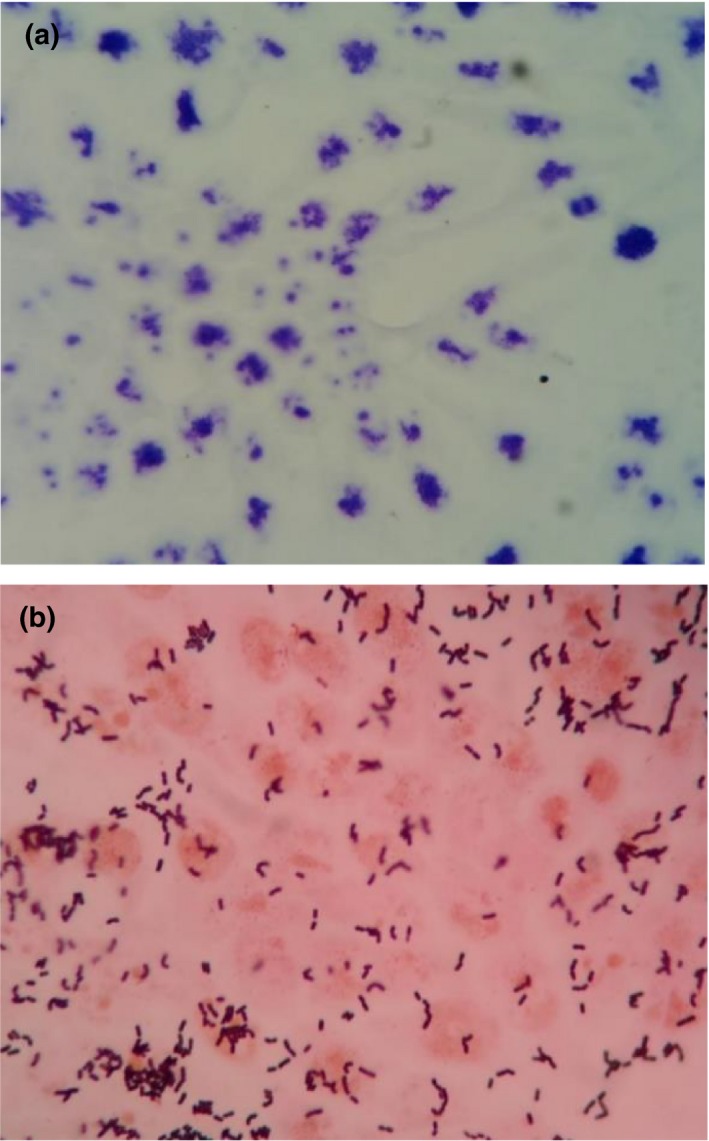
Adhesion of *Lactobacillus plantarum* to HT‐29 human colon adenocarcinoma cell line as indicated by Gram staining under a light microscope. (a) Untreated HT‐29 colon adenocarcinoma cell monolayer and (b) HT‐29 colon adenocarcinoma cell monolayer with *Lactobacillus plantarum* (magnification 1,000×)

**Figure 5:**
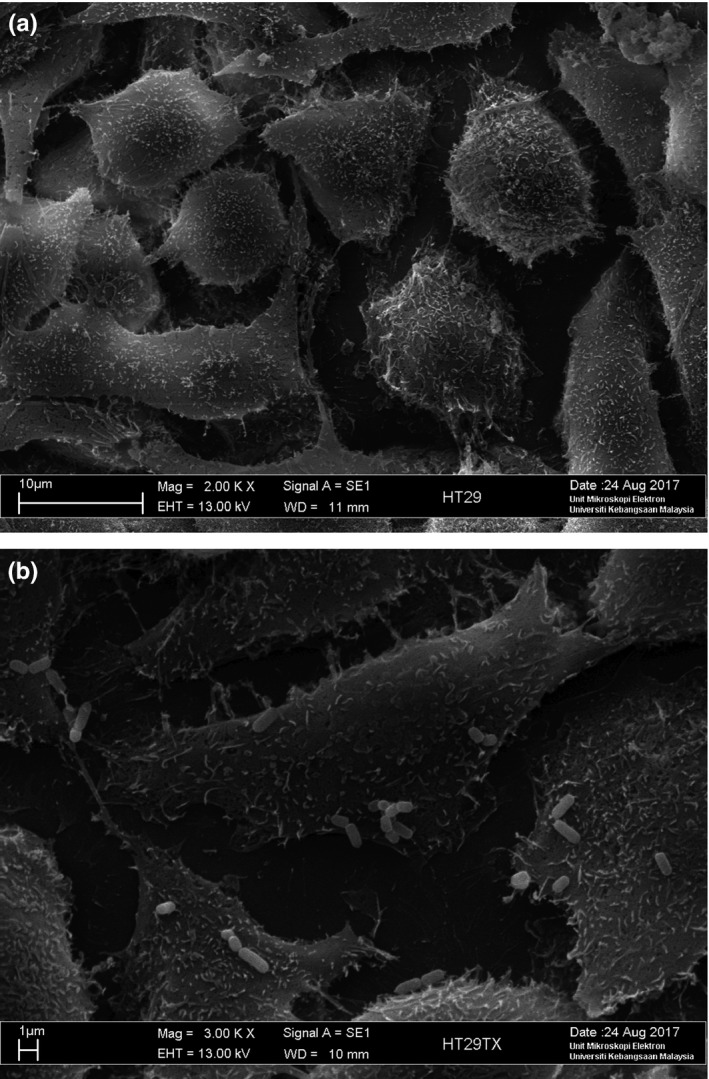
Scanning electron micrograph of (a) untreated HT‐29 colon adenocarcinoma cell line (magnification level 2,000×) (b) adhered *Lactobacillus plantarum* on HT‐29 colon adenocarcinoma cell line (magnification 3,000×)

Adhesion of lactobacilli has been deemed essential for exertion of beneficial probiotic effects in the large intestine (Maragkoudakis et al., 2006). In our study, adhesion of *L. plantarum* on HT‐29 cells was quantified (Figure 4) and showed an average of 159 ± 10 cells adhered to 100 HT‐29 epithelial cells (Table 4). Adhesion of *L. plantarum* to HT‐29 was further confirmed by SEM (Figure 5). Bacteria cells appeared in short chains or in pairs on the colon adenocarcinoma cells. *L. plantarum* showed strong adhesion on HT‐29 cell line. Jacobsen et al. (1999) classified bacterial adhesive properties into three categories: (1) nonadhesive when less than 40 cells adhered, (2) adhesive when there were 41–100 cells adhered, and (3) strongly adhesive when the number of adhered bacteria exceeds 100 cells.

**Table 4:** Adhesion of probiotic lactic acid bacteria to HT‐29 colon adenocarcinoma cell line

Bacteria	Adhesion Index on HT‐29 colon adenocarcinoma cell line
*Lactobacillus plantarum*	159.2 ± 10

In vitro adhesion of *L. plantarum* was monitored after 2 hr of incubation. The data are presented as mean ± standard deviation of bacteria adhered to cell line (*n* = 3).

Adherence of probiotic bacteria to colon epithelial limits colonization of pathogenic microorganisms and is able to modulate host immune system efficiently (Gopal et al., 2001; Wagner & Johnson, 2017). Previous studies by Oguntoyinbo and Narbad (2015) and Lee, Kim, Han, Eom, and Paik (2014) also showed that *L. plantarum* isolated from fermented cereal food and kimchi possessed strong adhesive properties on HT‐29 cells. The bacterial adhesion is most probably due to cell‐surface adhesive factors such as lectin/adhesion proteins of S‐layers, secreted lectin‐like bacteriocins, and lectin‐like complexes on lactobacilli and bifido BC walls (Lakhtin et al., 2006). According to Pretzer et al. (2005), in vitro adhesion of *L. plantarum* was also mediated by mannose components on the bacterial surface that bind to mannose receptor on the epithelial surface of human intestinal cells.

## 4 CONCLUSION

*Lactobacillus plantarum* isolated from tempoyak showed reasonably good probiotic properties including acid and bile salts tolerance, antioxidative, antiproliferative effects, and remarkable adhesion on HT‐29 cells. This was the first study to demonstrate probiotic potentials of lactic acid bacterium isolated from tempoyak and thus particularly useful as a reference for future developments of *L. plantarum* AN6 as a potential probiotic bacterium in functional food industry. In order to better apply the isolate as probiotic strain, in vivo experiments are needed to better understand the mechanisms of actions involved in its probiotic properties.

## ETHICAL REVIEW

This study does not involve any human or animal testing.

## CONFLICT OF INTEREST

The authors declare that they have no conflict of interest.
